# Frailty index predicts the risk of 17 health outcomes in distinct ways: prospective findings from the Moli-sani Study

**DOI:** 10.1093/ageing/afag091

**Published:** 2026-04-12

**Authors:** Francesca Bracone, Augusto Di Castelnuovo, Simona Costanzo, Alessandro Gialluisi, Marialaura Bonaccio, Mariarosaria Persichillo, Amalia De Curtis, Sara Magnacca, Teresa Panzera, Sabatino Orlandi, Antonio Cherubini, Chiara Cerletti, Maria Benedetta Donati, Giovanni de Gaetano, Licia Iacoviello

**Affiliations:** Research Unit of Epidemiology and Prevention, IRCCS Neurological Institute of Southern Italy NEUROMED, Pozzilli, Molise 86077, Italy; Research Unit of Epidemiology and Prevention, IRCCS Neurological Institute of Southern Italy NEUROMED, Pozzilli, Molise 86077, Italy; Research Unit of Epidemiology and Prevention, IRCCS Neurological Institute of Southern Italy NEUROMED, Pozzilli, Molise 86077, Italy; Department of Medicine and Surgery, University of Insubria, Varese, Lombardy, Italy; Research Unit of Epidemiology and Prevention, IRCCS Neurological Institute of Southern Italy NEUROMED, Pozzilli, Molise 86077, Italy; Department of Medicine and Surgery, LUM Giuseppe Degennaro University, Casamassima, Molise, Italy; Research Unit of Epidemiology and Prevention, IRCCS Neurological Institute of Southern Italy NEUROMED, Pozzilli, Molise 86077, Italy; Research Unit of Epidemiology and Prevention, IRCCS Neurological Institute of Southern Italy NEUROMED, Pozzilli, Molise 86077, Italy; Research Unit of Epidemiology and Prevention, IRCCS Neurological Institute of Southern Italy NEUROMED, Pozzilli, Molise 86077, Italy; Research Unit of Epidemiology and Prevention, IRCCS Neurological Institute of Southern Italy NEUROMED, Pozzilli, Molise 86077, Italy; Research Unit of Epidemiology and Prevention, IRCCS Neurological Institute of Southern Italy NEUROMED, Pozzilli, Molise 86077, Italy; Research Unit of Epidemiology and Prevention, IRCCS Neurological Institute of Southern Italy NEUROMED, Pozzilli, Molise 86077, Italy; Geriatrics and Geriatrics Emergency Care, Italian National Research Center on Aging (IRCCS-INRCA), Ancona, Italy; Department of Clinical and Molecular Sciences, Università Politecnica delle Marche, Ancona, Marche, Italy; Research Unit of Epidemiology and Prevention, IRCCS Neurological Institute of Southern Italy NEUROMED, Pozzilli, Molise 86077, Italy; Research Unit of Epidemiology and Prevention, IRCCS Neurological Institute of Southern Italy NEUROMED, Pozzilli, Molise 86077, Italy; Research Unit of Epidemiology and Prevention, IRCCS Neurological Institute of Southern Italy NEUROMED, Pozzilli, Molise 86077, Italy; Research Unit of Epidemiology and Prevention, IRCCS Neurological Institute of Southern Italy NEUROMED, Pozzilli, Molise 86077, Italy; Department of Medicine and Surgery, LUM Giuseppe Degennaro University, Casamassima, Molise, Italy

**Keywords:** frailty, neurodegenerative diseases, cardiometabolic risk, competing risks, ageing, mortality, older people

## Abstract

**Background:**

Frailty reflects systemic vulnerability and is a major public health concern in ageing. This study examined how frailty relates to risk of death, hospitalisation and major chronic diseases.

**Methods:**

We analysed data from 20 975 adults (≥35 years, 52% women) recruited in 2005–10 from the population-based Moli-sani Study (Italy) and followed for a median of 15 years. Frailty was assessed using a multidimensional 29-item frailty index (FI). Cox models accounting for competing risks estimated hazard ratios (HR) for 17 incident outcomes, adjusted for age, sex and common covariates, including social status indicators.

**Results:**

Frailty was associated with increased risk of several adverse outcomes. Per 1-SD increase in FI, the risk of type-2 diabetes and coronary heart disease rose by 82% (HR = 1.82; 95% confidence interval 1.73–1.92; 1541 events) and 33% (HR = 1.33; 1.23–1.44; 756). FI was also associated with an increased risk of Parkinson’s disease (HR = 1.25; 1.05–1.47; 158) and non-Alzheimer dementia (HR = 1.31; 1.11–1.54; 150). Cancer associations varied by site. FI also predicted hospitalisations for any cause (HR = 1.31; 1.28–1.34; 11 193), and all-cause mortality (HR = 1.35; 1.30–1.40; 2631). Analyses comparing frail and prefrail with fit categories, excluding early events, and stratified by sex showed consistent results, with indications of somewhat stronger associations among individuals younger than 65 years in certain outcomes.

**Conclusion:**

FI predicts a wide range of chronic diseases, especially cardiometabolic and neurodegenerative outcomes, and their negative consequences, including hospitalisation and mortality. These findings reinforce the importance of frailty assessment in preventive strategies, risk stratification and integrated surveillance in the general population.

## Key Points

- Frailty index predicts 17 health outcomes over 15 years in 20 975 Mediterranean adults aged 35+.
- Strongest links found with cardiometabolic diseases: 82% higher diabetes risk, 33% higher coronary disease risk.
- Frailty predicts Parkinson’s and non-Alzheimer dementia but not Alzheimer’s disease when accounting for competing mortality.
- Frailty shows stronger hazard ratios in adults under 65 than older adults for most outcomes.
- Cancer associations vary by site: increased lung cancer, decreased colorectal cancer, no link with breast/prostate cancers.

## Introduction

Frailty, conceptualised through the deficit-accumulation model of Rockwood and Mitnitski, quantifies biological ageing as the proportion of health deficits that an individual carry [1, 2]. Despite being a geriatric construct, frailty already affects ≈10%–15% of adults younger than 65 years [3]. Large population studies, including the Canadian Study of Health and Aging [4], and the China Health and Retirement Longitudinal Study [5] have consistently shown that higher frailty index (FI) values predict all-cause mortality and disability. Subsequent analyses revealed dose-dependent gradients for cardiovascular events, with a 29% higher CVD risk per 0.1-unit increment in the FI observed in a large Asian cohort [5] and for cardiometabolic multimorbidity in UK Biobank [6]. FI is also linked to incident type 2 diabetes, suggesting shared inflammatory–metabolic mechanisms [7].

Neurological endpoints have recently gained attention with regard to frailty: a meta-analysis demonstrated that frailty predicts Alzheimer disease, vascular dementia and all-cause dementia [8]. Moreover, a recent prospective study in a large UK cohort found that higher FI scores were associated with an increased risk of incident Parkinson’s disease (PD) [9]. In longitudinal cohorts of newly diagnosed Parkinson’s patients, baseline frailty has also been linked to subsequent dementia risk [10]. Although evidence on PD incidence is still emerging, prospective data from UK cohorts suggest a potential association between FI and incident PD [9].

Evidence on the link with oncological diseases is more heterogeneous: frailty is associated with increased total cancer mortality [11], but shows site-specific positive, null or inverse associations, particularly for colorectal cancer [12, 13].

Two approaches dominate frailty measurement. The frailty phenotype introduced by Fried, centred on sarcopenia-related criteria, captures a narrow physical domain [14]. In contrast, the deficit-accumulation FI used here integrates chronic diseases, symptoms and functional impairments, offering finer risk granularity and suitability for multi-system analyses [1, 2].

The FI has been associated with several negative outcomes, with a higher predictive validity than the frailty phenotype [15]. However, the majority of previous studies suffer from methodological limitations: the use of frailty categories does not allow to identify gradients/does-response effects while ignoring competing events—e.g. death when analysing dementia—biasing effect estimates [16].

The population-based Moli-sani Study provides an ideal setting to address these gaps [17]. The cohort offers deep baseline phenotyping, and comprehensive 15-year linkage to mortality, hospital discharge and disease registries. Crucially, the inclusion of adults aged ≥35 years allows us to explore the understudied phenomenon of frailty occurrence in middle aged individuals, recently associated with steeper hazard ratios than those observed in older adults [18].

By modelling frailty both as a continuous exposure and as categories (fit, pre-frailty and frailty) and applying cause-specific Cox models with competing-risk censoring to 17 endpoints analysed side-by-side, our study seeks to clarify how multisystem vulnerability translates into distinct disease trajectories and negative clinical outcomes. Subgroup analyses by sex and age will also be conducted, under the hypothesis that associations may differ across sex and age strata.

## Methods

### Study population

**Figure 1:**
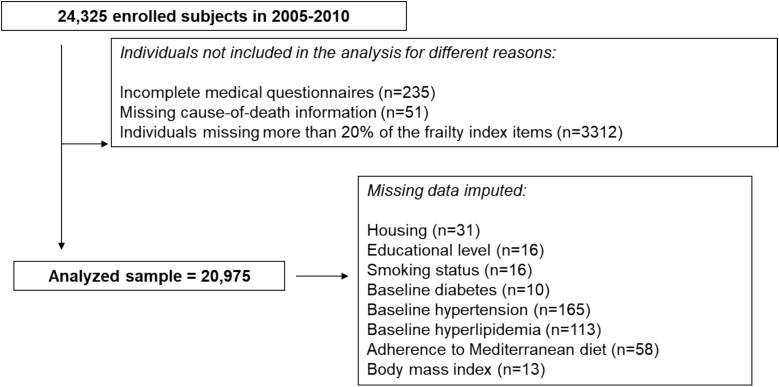
Flowchart of the study. The groups of removed participants (out of the 24 325 recruited at baseline) are overlaid. The final study sample cannot be calculated as a subtraction of the sum of eliminated groups out of the recruited individuals at baseline.

This analysis used data from the Moli-sani Study, a prospective cohort established between 2005 and 2010 (recruitment phase) in the Molise region of Southern Italy. The cohort includes 24 325 community-dwelling adults aged ≥35 years, aimed at investigating genetic and environmental determinants of vascular, cancer and neurodegenerative diseases. Participants were excluded if pregnant, unable to provide informed consent due to cognitive impairment, or affected by acute traumatic conditions (e.g. polytrauma, coma). Detailed methods have been reported elsewhere [19]. Participants lacking complete information from the medical questionnaire, or with missing data on cause of death or FI at baseline, were excluded from the present analysis. The selection process is illustrated in the study flowchart (Figure 1). The final sample available for analysis comprised 20 975 individuals.

### Frailty index

The FI was constructed following the deficit accumulation model originally developed by Rockwood and colleagues, which conceptualises frailty as a multidimensional state of vulnerability arising from the accumulation of health deficits [1, 2]. We adhered to the standardised 10-step procedure outlined by Theou *et al*. [20], specifically designed to guide the derivation of a FI from existing datasets in observational cohorts. This procedure provides reliable results independent of the source of data, provided a sufficient range of deficits is included in the FI [21].

A total of 29 baseline variables were selected following standard criteria: deficits had to be biologically plausible, age-related, linked to adverse outcomes, and neither too rare nor too common across ages. The full list is shown in Supplementary Table S1. Variables covered chronic diseases (e.g. coronary heart disease, cancer, diabetes, hypertension, kidney disease), symptoms (e.g. dyspnoea, fatigue), functional limitations (e.g. role limitations, pain) and self-rated or psychological health (e.g. general and mental health, depressive symptoms). Each item was scored from 0 (no deficit) to 1 (fully present). The FI was calculated for each participant as the ratio of observed deficits to total items, yielding a continuous score from 0 to 1; higher values indicated greater frailty. For categorical analyses, participants were classified as fit (FI < 0.10), pre-frail (0.10–0.19) or frail (≥ 0.20) [22]. In line with the recommendations by Theou *et al*. [20] individuals with more than 20% missing FI items were excluded from the analysis (*n* = 3312; Figure 1).

### Outcomes

Detailed definitions and ascertainment procedures for all outcomes (mortality, hospitalisation, neurodegenerative, cancer, vascular diseases and type 2 diabetes) are provided in the Supplementary Methods. Follow-up extended from cohort baseline (2005–10) until 31 December 2020, for hospitalisations, cancer, CVD and diabetes, and until 31 December 2022, for mortality and neurodegenerative outcomes, with participants who had a history of the corresponding condition at baseline excluded from the respective analyses.

### Covariates

At baseline, history of diabetes, hypertension and hyperlipidaemia were collected through structured interviews. For each condition, medication use was verified by inspecting drug packages brought by participants. A condition was recorded as present when both self-report and medication use were consistent. Height and weight were measured, and body mass index (BMI) calculated as kg/m^2^. Smoking was categorised as never, current or former (no smoking for ≥12 months). Leisure-time physical activity was expressed as daily energy expenditure (MET-hours/day) from sports, walking and gardening. Blood pressure was measured with an automated device (OMRON-HEM-705CP); three readings were taken on the non-dominant arm, and the mean of the last two was used. Socioeconomic status was assessed through education (≤8 vs ≥9 years) and housing (rented, one dwelling owned or multiple dwellings). Dietary intake over the previous year was estimated using the validated Italian version of the EPIC food frequency questionnaire [23]. Adherence to the traditional Mediterranean diet was evaluated using the Mediterranean Diet Score proposed by Trichopoulou and collaborators [24], with values ranging from 0 to 9 (maximum adherence).

### Statistics

Continuous variables were summarised as mean ± standard deviation (SD), and categorical variables as counts and percentages. Associations between the FI (per 1 SD) or frailty categories and incident outcomes were assessed using Cox proportional hazards models with time-on-study as the time scale and age adjustment. Follow-up started at baseline and ended at death, emigration, loss to follow-up (*n* = 355; 1.5%) or study end. For each outcome, follow-up continued until the event, death or censoring. When multiple outcomes within the same domain (neurological, oncological or cardiovascular) were possible, participants were censored at the first competing event in that domain. Cause-specific hazard ratios were estimated using cause-specific Cox models, suitable for etiologic interpretation. The proportional hazards assumption was verified with log(−log) plots and met in all cases. Participants with baseline history of a given disease were excluded from analyses of that outcome. Prostate and breast cancers were analysed in men and women, respectively. Incident dementia data were available for 76% of the cohort; ongoing data collection for the rest was unrelated to participant characteristics.

Potential confounders were defined a priori using a Directed Acyclic Graph built with the *dagitty* R package (v0.3-4; R 4.3.2). The minimal adjustment set included age (continuous), sex (binary), housing (rented, one or multiple dwellings), education (≤8 vs ≥9 years), smoking (never, former, current), Mediterranean diet score (continuous) and leisure-time physical activity (continuous) [25]. Sensitivity analyses excluded participants with ≤1 or ≤3 years of follow-up to assess potential reverse causation. Subgroup analyses were stratified by sex and age (35–64.9 vs ≥65 years), and interaction terms (FI × sex; FI × age group) tested for effect modification. Missing data were handled using multiple imputation (ten datasets) with SAS PROC MI and PROC MIANALYZE. All analyses were performed with SAS/STAT 9.4 (SAS Institute Inc., Cary, NC, USA).

## Results

The FI ranged from 0 to 0.66, showing a slightly right-skewed but near-symmetric distribution (Supplementary Fig. S1). The mean FI was 0.17 (SD 0.08) and the median 0.16. Only two participants had FI ≥ 0.55, consistent with end-stage frailty as defined by Kim [22]; given their small number, they were included in the *frailty* group (FI ≥ 0.20). Table 1 shows baseline characteristics by frailty category: fit (FI < 0.10, 21.9%), pre-frail (0.10–0.19, 48.9%) and frail (≥0.20, 29.3%). As expected, frailty followed a clear gradient—frailer participants were older, had more comorbidities (CVD, cancer, diabetes, hypertension, hypercholesterolemia), higher BMI and lower physical activity. Women and individuals with lower education were more common in the frailer groups, with the largest differences in the frail category. These trends were consistent across age strata (35–64.9 and ≥ 65 years; Supplementary Table S2).

**Table 1:** Baseline characteristics of the studied population, overall and by frailty categories

	All	Fit	Pre-frailty	Frailty
N of individuals (*n*, %)	20 975 (100%)	4582 (21.9%)	10 249 (48.9%)	6144 (29.3%)
Frailty Index (points; mean (SD))	0.17 (0.08)	0.07 (0.02)	0.15 (0.03)	0.27 (0.05)
Frailty Index (points, min-max)	0–0.664	0–0.099	0.100–0.199	0.200–0.664
Women (%)	51.7	45.2	48.1	62.4
Age (years)	55 ± 11	48 ± 9	54 ± 10	61 ± 11
Cardiovascular disease (%)	5.2	0.29	2.00	14.2
Cancer (%)	3.2	0.4	2.4	6.8
Diabetes (%)	9.0	0.7	6.9	18.6
Hypertension (%)	54.4	19.6	55.1	79.3
Hyperlipidaemia (%)	30.6	10.2	30.2	46.6
Educational level (%)				
Up to lower secondary	48.7	33.8	46.9	62.8
Upper secondary	51.3	66.2	53.1	37.2
Housing categories (%)				
Rented	8.9	8.6	8.8	9.4
1 Dwelling ownership	81.7	83.7	81.7	80.2
>1 Dwelling ownership	9.3	7.7	9.5	10.4
Smoking status (%)				
Non-smokers	48.3	50.3	45.5	51.6
Current	24.0	26.5	25.8	19.1
Former	27.7	23.3	28.7	29.3
Leisure-time physical activity (MET-h/day)	3.5 ± 3.9	4.0 ± 4.0	3.6 ± 4.0	2.9 ± 3.7
Body mass index (kg/m^2^)	27.9 ± 4.7	25.7 ± 3.7	27.7 ± 4.3	29.8 ± 5.2
Mediterranean Diet Score (points)	4.4 ± 1.6	4.3 ± 1.6	4.4 ± 1.7	4.4 ± 1.6

Continuous and categorical variables are expressed as mean values and standard deviation (SD) or percentage, respectively. All differences across frailty categories had *P* < .0001, except for the Mediterranean Diet Score (*P* = .0014).

### Frailty and clinical outcomes

Supplementary Fig. S2 shows spline curves for the association between continuous FI and mortality. The relationship was consistently linear, with hazard ratios for overall and cause-specific mortality increasing steadily with higher FI and no sign of non-linearity. Therefore, FI was treated as a linear exposure in subsequent analyses.

**Figure 2:**
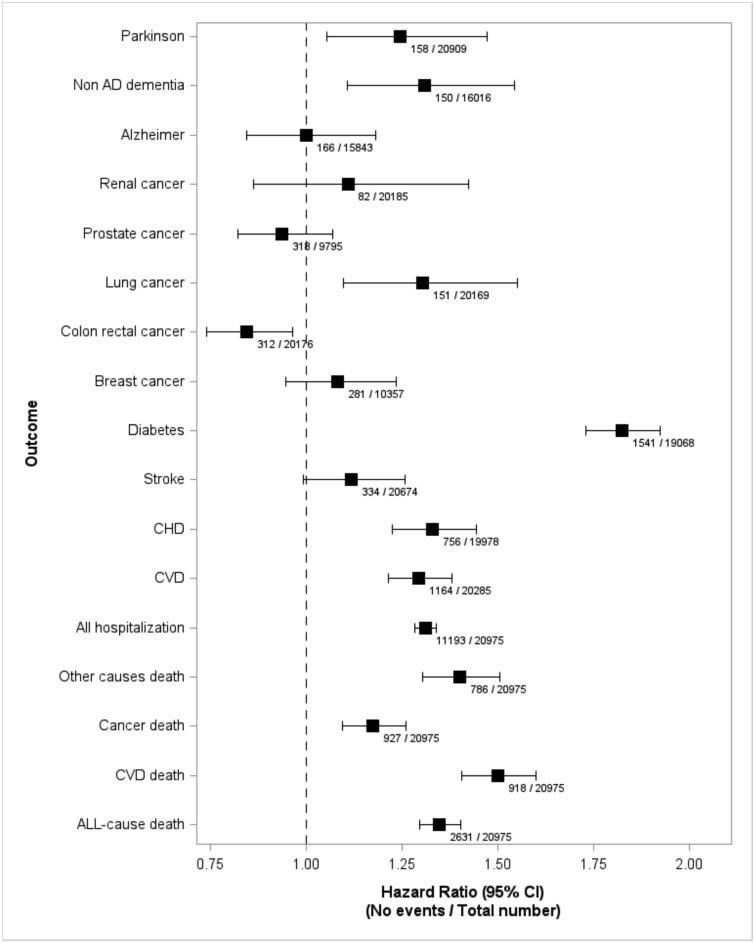
Multivariable hazard ratios for a 1-standard deviation increase in the frailty index across different outcomes. Analyses account for competing risks and are adjusted for age, sex, smoking status, educational level, housing status, adherence to the Mediterranean diet, and leisure-time physical activity. Horizontal bars represent 95% confidence intervals. Numbers at the lower right of each outcome indicate the number of events during follow-up and the baseline population at risk.

Figure 2 shows the associations between FI (per 1-SD increase) and multiple incident outcomes, expressed as hazard ratios from multivariable Cox models, adjusted for age, sex, smoking status, educational level, housing status, adherence to the Mediterranean diet and leisure-time physical activity. Numbers on the right of each panel indicate the number of events and participants at risk at baseline. FI was positively associated with higher risk of hospitalisation (HR = 1.31; 95% CI: 1.28–1.34; rate = 606.1 events per 10 000 person-years (py)), all-cause mortality (HR = 1.35; 1.30–1.40; 86.1 per 10 000 py), and cardiovascular mortality (HR = 1.50; 1.41–1.60; 30.0 per 10 000 py). Among non-fatal outcomes, the strongest associations were observed for diabetes (HR = 1.82; 1.73–1.92; 62.6 per 10 000 py) and coronary heart disease (HR = 1.33; 1.23–1.44; 30.7 per 10 000 py).

For neurological outcomes, FI was associated with increased risk of PD (HR = 1.25; 1.05–1.47; 5.3 per 10 000 py) and non-Alzheimer dementia (HR = 1.31; 1.11–1.54; 5.0 per 10 000 py), but not with Alzheimer’s disease.

Regarding cancer, higher FI was linked to increased incidence of lung cancer (HR = 1.30; 1.10–1.55; 5.9 per 10 000 py), decreased risk of colorectal cancer (HR = 0.84; 0.74–0.96; 12.3 per 10 000 py), but no association with breast, prostate or renal cancers was observed. Cancer mortality was increased with frailty (HR = 1.17; 1.10–1.26; 30.3 per 10 000 py).

**Figure 3:**
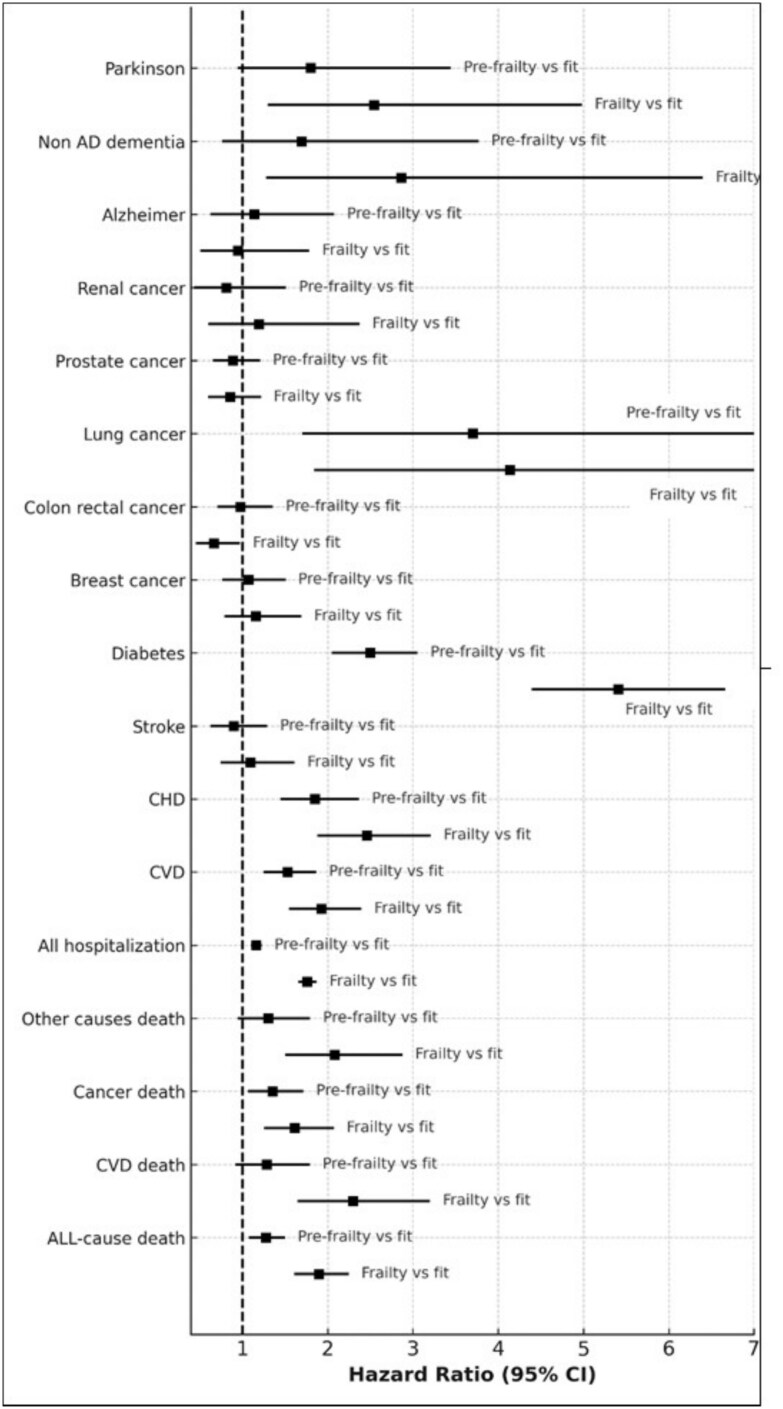
Multivariable hazard ratios for frailty categories across different outcomes. Analyses account for competing risks and are adjusted for age, sex, smoking status, educational level, housing status, adherence to the Mediterranean diet, and leisure-time physical activity. Horizontal bars represent 95% confidence intervals. The fit group was the referent category, and HRs are reported for pre-frailty and frailty *versus* fit.

Similar results were observed when frailty was analysed in categories (Figure 3). Associations were consistently stronger for the comparison of frailty versus fit, while the pre-frailty condition also remained associated with higher risk of lung cancer, diabetes, coronary heart disease, cardiovascular disease, hospitalisation and death.

### Subgroups and sensitivity analyses

**Figure 4:**
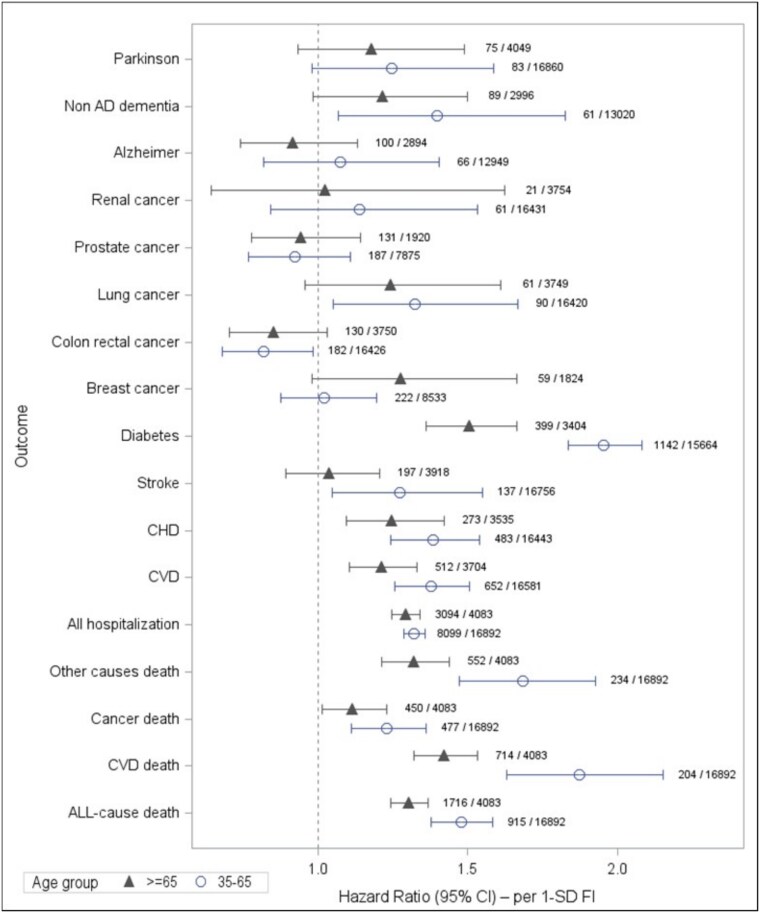
Sensitivity analyses of multivariable hazard ratios associated with a 1-standard deviation increase in the Frailty Index (FI), stratified by age group (35–64.9 and ≥ 65 years). Analyses account for competing risks and are adjusted for age (as a continuous variable), smoking status, educational level, housing status, adherence to the Mediterranean diet, and leisure-time physical activity. For each outcome, hazard ratios are shown for individuals aged 35–64.9 years (black square markers) and ≥ 65 years (grey triangle markers). Horizontal bars represent 95% confidence intervals. Numbers at the lower right of each outcome indicate the number of events during follow-up and the number of individuals at risk at baseline in each analytic subsample.

In age-stratified models (Figure 4), frailty–cancer associations were similar across age groups, while for all other outcomes hazard ratios were higher in participants aged 35–64.9 years, most notably for incident diabetes.

Supplementary Fig. S3 reports sensitivity analyses excluding participants with ≤1 and ≤ 3 years of follow-up. Associations between FI and outcomes remained essentially unchanged, indicating that early events or reverse causation did not account for the main results.

Supplementary Fig. S4 presents sex-stratified analyses. Associations between FI and outcomes were broadly similar in men and women, with no major differences. The only exception was all-cause hospitalisation, where the HR per 1-SD increase in FI was slightly higher in men (p for interaction <0.001). This difference is likely not clinically relevant and may reflect the large number of events and resulting higher statistical power.

## Discussion

In a single, large population-based cohort of Mediterranean adults with long follow-up, we examined baseline frailty in relation to 17 incident outcomes—cardiovascular, oncologic, neurologic, hospitalisation and death for various causes—using time-to-event models with competing risks. To our knowledge, analyses covering such a broad set of outcomes within one dataset are uncommon. The distribution of frailty categories in our cohort (22% fit, 49% pre-frail, 29% frail) is consistent with that reported in other population-based studies [22, 26].

The FI and categories were consistently linked to incident events across all domains, with similar or stronger associations in participants aged 35–64.9 than in older adults, indicating that FI captures vulnerability already in midlife. Frailty emerged as a strong marker of overall vulnerability predicting a wide range of outcomes in Mediterranean adults. The steepest gradients were seen for lung cancer and cardiometabolic diseases: diabetes and coronary heart disease increased by about 80% and 55% per 1-SD rise in FI, closely matching estimates from the HRS and UK Biobank [6, 27].

Frailty also predicted neurodegenerative outcomes, though patterns differed by subtype. The excess risks for Parkinson’s disease and non-Alzheimer dementia align with results from the Swedish Twin Registry [28] and a recent meta-analysis [29], both identifying frailty as an upstream factor in vascular and Lewy-body pathology. In contrast, our study, like others [30, 31], found no association with Alzheimer’s disease after accounting for competing events such as death and PD. This supports the view that Alzheimer’s pathology may be less affected by multisystem deficit accumulation than other late-life brain disorders.

Cancer results were heterogeneous, and in line with previous observations: positive for lung [32], inverse for colorectal [12, 13] and null for breast, prostate and renal cancers [33].

The FI includes several chronic conditions (such as diabetes, cardiovascular disease and cancer) that also appear as study outcomes. While we excluded participants with baseline disease from each respective outcome analysis, some degree of conceptual overlap remains, as the FI captures overall deficit accumulation that includes comorbidities. However, this feature is inherent to the deficit-accumulation model: frailty reflects systemic vulnerability arising from multiple health deficits, not just the presence of specific diseases. The fact that a higher FI predicts incident conditions beyond those already present supports its utility as a risk stratification tool in clinical practice, where identifying vulnerable individuals across multiple disease trajectories is often more relevant than predicting single outcomes in isolation [22].

Moreover, the FI offers practical advantages over disease-specific biomarkers: it is low-cost, non-invasive, and can be constructed from routinely collected clinical data without additional laboratory testing. While individual effect sizes may appear modest, the FI simultaneously predicts multiple adverse outcomes in a single assessment, making it particularly valuable for population-level risk stratification and primary prevention strategies where screening large numbers of individuals with multiple tests would be impractical or cost-prohibitive. In this context, even modest associations translate into substantial population health impact when applied broadly across the general adult population [2, 22, 45].

### Biological mechanisms underlying frailty

The steady increase in risk across outcomes with higher frailty levels supports the idea that frailty reflects a systemic inflammatory–metabolic state. Chronic low-grade ‘inflammaging’, marked by elevated IL-6 and TNF-α, mitochondrial dysfunction, and reduced autophagy, accelerates endothelial, musculoskeletal and neuronal damage [34]. Experimental data further implicate DNA-damage signalling and cellular senescence in dopaminergic neurons, potentially explaining the selective link with Parkinson’s disease [35]. Frailty has been associated with hypoalbuminemia [36], a risk factor for mortality in the studied population [37], and it often coexists with polypharmacy [38], which in turn has been linked, in the same population, to increased risks of hospitalisation and death [39].

Beyond specific diseases, frailty strongly predicted first hospitalisation and all-cause mortality. A 35% rise in mortality per 1-SD increase in FI matched estimates from the Austrian Health Interview Survey (HR = 1.33 per 0.1 FI increase) [40], and with pooled estimates from meta-analyses [29], underscoring the external validity of our findings across different populations. Sensitivity analyses excluding events within the first 1 or 3 years produced nearly identical results, reducing the chance that reverse causation or pre-existing disease drove the associations.

### Explaining cancer heterogeneity

The differing cancer results show that frailty is not a uniform risk factor for all cancers. Lung cancer was positively associated with FI, consistent with evidence that systemic inflammation fosters tumour development and that frailer individuals accumulate more smoking-related damage [32]. The inverse association with colorectal cancer likely reflects competing-risk depletion: frailer adults may die earlier from cardiovascular or pulmonary causes, leaving the at-risk pool before colorectal cancer can occur [12, 13]. Screening bias may also contribute: frail individuals are less likely to undergo colonoscopy and may die from other causes before asymptomatic colorectal lesions become clinically apparent [41]. The result is an apparent protective association that vanishes when deaths are treated as competing events.

Overall, the variability across cancer types likely mirrors the biological and clinical heterogeneity of cancer itself.

### Effect modification by age and sex

For several non-cancer outcomes, hazard ratios tended to be higher in adults aged 35–64.9 years, suggesting that frailty, when it arises prematurely, may be more detrimental than when it occurs as part of expected ageing [42]. This pattern supports the concept of early-onset frailty—a deficit-accumulation phenotype that emerges ahead of the typical ageing trajectory and magnifies downstream risk [18, 43, 44]. This finding is consistent with evidence that multimorbidity and frailty are closely related but distinct constructs: most frail individuals have multiple chronic conditions, yet frailty typically develops as diseases accumulate over time. In younger adults, a rising FI may capture an active phase of accelerated deficit accumulation, marking individuals who are ageing faster than expected and are at high risk of developing further chronic diseases [45].

From a public health perspective, detecting frailty in working-age adults offers an opportunity for early intervention before disability occurs and chronic diseases multiply. This has clear economic implications: preventing premature frailty could reduce healthcare costs, avoid early workforce exit, and maintain productivity in a population that is still decades away from expected retirement [46].

Sex did not materially alter effect sizes, suggesting that frailty embodies biological damage accruing across social strata; nevertheless, future work should explore whether resource-based resilience modulates progression from subclinical to clinical disease.

### Strengths and limitations

A major strength of this study is its long follow-up, allowing assessment of frailty’s long-term associations with a wide range of outcomes.

Methodologically, strengths include: (i) modelling frailty mainly as a continuous variable to retain dose–response information, with complementary categorical analyses; (ii) evaluating 17 fatal and non-fatal outcomes within a unified framework and (iii) using cause-specific Cox models that account for competing events, providing interpretable etiologic estimates.

Limitations arise from the observational design. Despite careful adjustment, residual confounding and selection bias cannot be ruled out. In particular, although we adjusted for education and housing status, our study lacked a recognised measure of area-based or individual deprivation, which might have left some aspects of socioeconomic status unmeasured. Measurement error is possible, particularly for self-reported data. Frailty, lifestyle, comorbidities, and other covariates were recorded only at baseline, preventing assessment of changes or trajectories over time and possibly introducing regression-dilution bias. The cohort’s regional composition (Southern Italy) may limit generalizability, warranting replication in other populations.

To reduce misclassification, major diagnoses were cross-checked with facility information, and medication use verified through drug packaging. While this improved accuracy, some self-report bias remains. These are common challenges in large-scale epidemiological research and should be considered when interpreting the findings.

## Conclusions and future directions

In this study, deficit-accumulation frailty was linked to multiple adverse outcomes, with two main observations: stronger associations at younger ages and outcome-specific patterns evident after accounting for competing risks. From a public-health perspective, these results support incorporating FI screening into prevention programs to better target lifestyle or pharmacological interventions. Future research integrating molecular data may clarify whether frailty’s biological basis represents a modifiable cause or a downstream marker, and whether targeted anti-inflammatory or geroscience-based strategies can slow biological ageing.

## Supplementary Material

afag091_Supplemental_File
