# Supplementary material for: Frailty index predicts the risk of 17 health outcomes in distinct ways: prospective findings from the Moli-sani Study
Source: Age Ageing. 2026 Apr 12;55(4):afag091. doi: 10.1093/ageing/afag091 (PMC13071405; doi:10.1093/ageing/afag091)
Supplement: afag091_Supplemental_File [file afag091_supplemental_file.docx]

**Frailty Index Predicts the Risk of 17 Health Outcomes in Distinct Ways: Prospective Findings from the Moli-sani Study**

**Contents list of Supplementary Data**

**Appendix 1**

Methods - Outcomes assessment

**Appendix 2**

Supplementary tables S1-S2

Supplementary figures S1-S2-S3-S4

**Appendix 3**

Moli-sani Study Investigators

**Appendix 1**

**Supplementary methods - Outcomes assessment**

*All-cause and cause-specific mortality*

The Moli-sani Study cohort was followed up for mortality from March 2005 through December 31, 2022, via deterministic linkage with the regional ReNCaM register (nominative register of causes of death). Cause-specific mortality was validated using official Italian death certificates (ISTAT form) and coded according to the International Classification of Diseases, ninth revision (ICD-9). Cardiovascular disease (CVD) mortality was defined as deaths due to diseases of the circulatory system (ICD-9 codes 390–459); among these, cerebrovascular deaths were identified using codes 430–438, while ischemic heart disease (IHD) deaths corresponded to codes 410–414 and 429. Cancer-related deaths were defined when the underlying cause was coded as ICD-9 140–208. All remaining non-CVD and non-cancer causes of death were grouped as “other causes”.

*Hospitalization for any cause*

The Italian healthcare system follows a single-payer system, and it is based on information reported in the regional register of hospital discharge records, which includes all hospitalizations of all citizens residing in a given region, both in private and public national hospitals. A hospitalization was defined as any length of stay of at least 24 h in a hospital, clinic, emergency room or other similar facility. If a patient was transferred to another hospital or facility, this was considered a single hospitalization. Hospitalizations for the following conditions were excluded: pregnancy complications, childbirth, rehabilitation, and chemotherapy and/or radiotherapy (i.e., elective day care in a hospital-based unit). Incidence was defined as the first occurrence of a hospitalization for any cause or cause-specific admission (S1). The Moli-sani Study cohort was followed up for any hospitalization until 31st December 2020.

*Neurodegenerative outcomes*

Ascertainment of the Parkinson’s disease status was carried out through linkage with Electronic Health Records databases using fiscal code of each participant as unique identifier. The Molise regional drug prescription registry was first used to identify patients starting PD therapy during the follow-up. According to the national health service, anyone prescribed a drug is entered into a specific registry maintained by the local health authority. Only patients with chronic use of medications with Anatomical Therapeutic Chemical (ATC) classification N04XX (anti-Parkinson drugs) were considered. A similar method for the identification of PD cases has been used in other studies (e.g., ref. 45). Then, drug registry identification of cases was integrated through the regional registry of hospital discharge records. Hospitalization was defined as any stay lasting ≥24 h in any hospital, clinics, emergency room, or similar, be it private or public. Hospitalizations showing an International Classification of Diseases, 9th revision (ICD-9) code for PD (332.0) in primary and secondary diagnoses were classified as incident cases. The earliest date indicating a PD status in the abovementioned registries was used to establish the onset of PD. Three independent neurologists with experience in movement disorders further validated PD diagnoses by combining information on anti-parkinsonian drugs in use (levodopa, dopamine agonists, MAO-B inhibitors) and hospital discharge records. Further details on the definition of PD are available elsewhere (S1). Incident cases of AD and other dementias, including frontotemporal dementia, Lewy body dementia, vascular dementia, and unspecified dementia, were identified through record linkage with medical registers from two major regional specialized centres for cognitive disorders. A team of neurologists validated these diagnoses using clinical data from outpatient visits, disease certifications, neurocognitive assessments, and neuroimaging tests such as MRI, CT, Amyloid-PET, and FDG-PET. The Moli-sani Study cohort was followed up for PA and AD until 31st December 2022. Individuals with a personal history of PD (n=66) or AD (n=27) at baseline were excluded from the analyses of PD and AD outcomes, respectively.

*Cancer outcomes*

Incident cases of cancer [breast (BC), prostate (PC), colorectal (CRC), lung (LC) and renal (RNC)] were ascertained by direct linkage with hospital discharge forms according to the ICD-9-CM code (BC: 174, PC:185, CRC: 153-154, LC: 162, PNC: 157, RNC: 189). Events were validated through medical records when the cancer was mentioned in the diagnosis and was confirmed by histological reports. Furthermore, fatal cases of cancer were ascertained by direct linkage with death registry (ReNCaM registry) if death certificates presented specific ICD-9 codes as the underlying cause of death (BC: 174, PC:185, CRC: 153-154, LC:162, PNC: 157, RNC: 189.

A clinical adjudication committee reviewed all medical data. The Moli-sani Study cohort was followed up for cancer events until 31st December 2020. Individuals with a history of any type of cancer at baseline (n=669) were excluded from the analysis of cancer outcomes.

*Cardiovascular outcomes*

Fatal and nonfatal incident cases of CHD, myocardial infarction, coronary revascularization and sudden death for unspecified cardiac event) and cerebrovascular disease that occurred in the cohort during follow-up were ascertained by linkage of the study cohort to the hospital discharge files and to the regional ReNCaM registry and death certificates (ISTAT form), by using the ICD-9. For CHD, ICD 9 codes 410-414 and/or reperfusion procedure (ICD-9 codes 36.0-36.9) and for cerebrovascular disease, ICD9 codes 430-432, 434, 436-438 or procedure codes for carotid revascularization (ICD 9 code 38.12) were considered. Suspected CHD deaths were identified when ICD-9 codes 410-414 or 798 and 799 were reported as the underlying cause of death or codes 250, 401-405, 420-429 as the underlying cause of death, associated with codes 410-414 as a secondary cause of death. Suspected cerebrovascular deaths were identified when ICD 9 codes 430-438 were reported as the underlying, antecedent, or direct cause of death. All events were validated using procedures of the AHA, WHF, ESC, CDC and NHLBI for epidemiology and clinical research studies ref. The Moli-sani Study cohort was followed up for cardiovascular events until 31st December 2020. Individuals with a personal history of CHD (n=690) or cerebrovascular disease (n=301) at baseline were excluded from the analyses of CHD and cerebrovascular outcomes, respectively.

*Incident type 2 diabetes*

Incident cases of type 2 diabetes mellitus (T2DM) were identified through individual-level deterministic record linkage with three regional sources: the hospital discharge records (HDRs), the ReNCaM mortality register, and the drug prescription register. The HDRs, which include all hospital admissions for residents of the Molise region, provided diagnostic information coded according to ICD-9; incident cases of T2DM were identified when the codes 250.x or 250.xx (excluding type 1 diabetes) appeared as a primary or secondary diagnosis in hospitalizations occurring between 2006 and 31st December 2020. In the ReNCaM register, T2DM was considered incident when ICD-9 code 250 was reported as either the underlying or a secondary cause of death. Additionally, the drug prescription registry was used to identify incident cases based on the Anatomical Therapeutic Chemical (ATC) classification system. Individuals were classified as having incident T2DM if, during follow-up, they were chronically treated with glucose-lowering medications (ATC code A10), defined as having an annual Defined Daily Dose (DDD) ≥110, corresponding to at least 30% of the expected DDD for chronic therapy. Information on the type of drug, quantity, and date of first dispensing was available from 2006 to 2020. Individuals receiving diabetic drug therapy with an annual DDD below this threshold or with inconsistent treatment during follow-up were categorized as being in occasional therapy and were not considered as incident cases. Individuals with a history of type 2 diabetes at baseline (n=1,076) were excluded from the analysis of diabetes outcome.

**References**

S1. Costanzo S, Di Castelnuovo A, Panzera T, De Curtis A, Falciglia S, Persichillo M, Cerletti C, Donati MB, de Gaetano G, Iacoviello L; Moli-sani Investigators. Polypharmacy in Older Adults: The Hazard of Hospitalization and Mortality is Mediated by Potentially Inappropriate Prescriptions, Findings From the Moli-sani Study. Int J Public Health. 2024 Oct 24;69:1607682. doi: 10.3389/ijph.2024.1607682. PMID: 39513180; PMCID: PMC11540657.

S2. Gialluisi A, De Bartolo MI, Costanzo S, Belvisi D, Falciglia S, Ricci M, Di Castelnuovo A, Panzera T, Donati MB, Fabbrini G, de Gaetano G, Berardelli A, Iacoviello L. Risk and protective factors in Parkinson's disease: a simultaneous and prospective study with classical statistical and novel machine learning models. J Neurol. 2023 Sep;270(9):4487-4497. doi: 10.1007/s00415-023-11803-1. Epub 2023 Jun 9. PMID: 37294324.

**Appendix 2**

**Supplementary Tables**

**Table S1**. **List of the 29 Items Included tn the Frailty Index and their Scoring**

| Items | Scoring |
| --- | --- |
| Atrial fibrillation | No=0, Yes=1 |
| Asthma | No=0, Yes=1 |
| Cerebrovascular disease | No=0, Yes=1 |
| Coronary heart disease | No=0, Yes=1 |
| Colitis or inflammatory bowel disease | No=0, Yes=1 |
| Esophageal, stomach or duodenal disease | No=0, Yes=1 |
| Hypercholesterolemia | No=0, Yes=1 |
| History of depression | No=0, Yes=1 |
| Hypertension | No=0, Yes=1 |
| Inflammatory arthritis | No=0, Yes=1 |
| Kidney disease | No=0, Yes=1 |
| Liver disease | No=0, Yes=1 |
| Any type of cancer | No=0, Yes=1 |
| Osteoporosis (untreated) | No=0, Yes=1 |
| Osteoporosis (treated) | No=0, Yes=1 |
| Pancreas, biliary or bladder disease | No=0, Yes=1 |
| Prostate disease | No=0, Yes=1 |
| Chronic pulmonary disease | No=0, Yes=1 |
| Type 2 diabetes mellitus | No=0, Yes=1 |
| Thyroid disease | No=0, Yes=1 |
| Overweight or obesity | No=0, Yes=1 |
| Shortness of breath during normal activity | No=0, Yes=1 |
| Shortness of breath while sleeping | No=0, Yes=1 |
| Orthopnea (needs two pillows) | No=0, Yes=1 |
| General health (poor) | No=0, Yes=1 |
| Physical functioning limitation | No=0, Yes=1 |
| Bodily pain (severe) | No=0, Yes=1 |
| Mental health problems | No=0, Yes=1 |
| Emotional role limitation | No=0, Yes=1 |

**Table S2 Baseline Characteristics of The Study Population by Frailty Categories, Stratified by Age Group (35–64.9 And ≥65 Years)**

|  | **Individuals aged 35-64.9 years** | | | **Individuals aged ≥65 years** | | |
| --- | --- | --- | --- | --- | --- | --- |
| N of individuals (n, %) | 4377 (25.9%) | 8705 (51.5%) | 3810 (22.6%) | 205 (5.0%) | 1544 (37.8%) | 2334 (57.2%) |
| Frailty Index (points; mean (SD)) | 0.07 (0.02) | 0.15 (0.03) | 0.26 (0.05) | 0.08 (0.02) | 0.16 (0.03) | 0.28 (0.06) |
| Frailty Index (points, min-max) | 0.0-0.1 | 0.1-0.2 | 0.2-0.5 | 0.0-0.1 | 0.1-0.2 | 0.2-0.7 |
| Women (%) | 46.1 | 50.0 | 65.6 | 25.9 | 37.8 | 57.1 |
| Age (years) | 46.5 (7.0) | 50.4 (7.6) | 54.4 (6.9) | 70.3 (4.4) | 71.1 (4.9) | 72.7 (5.5) |
| Cardiovascular disease (%) | 0.3 | 1.4 | 9.3 | 0.5 | 5.6 | 22.2 |
| Cancer (%) | 0.4 | 1.9 | 5.8 | 1.0 | 4.7 | 8.4 |
| Diabetes (%) | 0.6 | 6.0 | 16.5 | 2.5 | 11.9 | 22.1 |
| Hypertension (%) | 17.6 | 50.4 | 72.4 | 60.8 | 82.0 | 90.7 |
| Hyperlipidaemia (%) | 10.3 | 30.6 | 48.0 | 9.4 | 27.7 | 44.4 |
| Educational level (%) |  |  |  |  |  |  |
| Up to lower secondary | 32.7 | 44.0 | 55.2 | 56.1 | 63.2 | 75.2 |
| Upper secondary | 67.3 | 56.0 | 44.8 | 43.9 | 36.8 | 24.8 |
| Housing categories (%) |  |  |  |  |  |  |
| Rented | 8.7 | 9.2 | 10.8 | 5.4 | 6.5 | 7.1 |
| 1 dwelling ownership | 83.9 | 81.9 | 78.8 | 79.0 | 81.0 | 82.6 |
| >1 dwelling ownership | 7.4 | 8.9 | 10.4 | 15.6 | 12.6 | 10.3 |
| Smoking status (%) |  |  |  |  |  |  |
| Non-smokers | 50.5 | 45.3 | 47.8 | 44.9 | 46.6 | 57.8 |
| Current | 22.5 | 26.6 | 26.9 | 38.5 | 40.4 | 33.1 |
| Former | 26.9 | 28.0 | 25.3 | 16.6 | 13.0 | 9.0 |
| Leisure-time physical activity (MET-h/day) | 3.9 (3.9) | 3.5 (3.8) | 2.8 (3.6) | 5.9 (4.6) | 4.6 (4.8) | 3.0 (4.0) |
| Body mass index (kg/m^2^) | 25.7 (3.7) | 27.7 (4.4) | 29.9 (5.3) | 26.4 (3.3) | 27.9 (4.1) | 29.6 (4.9) |
| Mediterranean Diet Score (points) | 4.3 (1.6) | 4.4 (1.7) | 4.4 (1.6) | 4.9 (1.7) | 4.6 (1.6) | 4.4 (1.6) |

Continuous and categorical variables are expressed as mean values and standard deviation (SD) or percentage, respectively. All differences across frailty categories showed P<0.0001 in both age groups, except for housing in individuals aged ≥65 years (P= 0.0045)

**Supplementary Figure legend**

**Figure S1.** Histogram showing the distribution of the Frailty Index at baseline in the Moli-sani population. The x-axis represents frailty scores, while the y-axis indicates the percentage of individuals in each bin. Summary statistics (mean, median, minimum, maximum, range, standard deviation, and skewness) are displayed in the inset box.

**Figure S2.** Restricted cubic spline analysis of the association between the Frailty Index (FI), treated as a continuous exposure, and mortality. Panels a to d show all-cause, cardiovascular, cancer-related, and other-cause mortality, respectively. Knots were placed at the 5th, 50th, and 95th percentiles of the FI distribution. The reference value was set at FI = 0.0. Dotted lines indicate 95% confidence intervals.

**Figure S3.** Sensitivity analyses of multivariable hazard ratios for a 1-standard deviation increase in the frailty index across different outcomes. Analyses adjusted for age, sex, smoking status, educational level, housing status, adherence to the Mediterranean diet, and leisure-time physical activity and competing risks were accounted for in domain-specific outcomes. For each outcome, hazard ratios are reported for the full cohort (black square markers), and after excluding individuals with ≤1 year (grey circle markers) and ≤3 years (light gray triangle markers) of follow-up. Horizontal bars represent 95% confidence intervals. Numbers at the lower right of each outcome indicate the number of events during follow-up and the baseline population at risk in each analytic subsample.

**Figure S4.** Sensitivity analyses of multivariable hazard ratios for a 1-standard deviation increase in the frailty index in men and women. Analyses adjusted for age, smoking status, educational level, housing status, adherence to the Mediterranean diet, and leisure-time physical activity and competing risks were accounted for in domain-specific outcomes. For each outcome, hazard ratios are reported for women (black square markers), and men (gray triangle markers). Horizontal bars represent 95% confidence intervals. Numbers at the lower right of each outcome indicate the number of events during follow-up and the baseline population at risk in each analytic subsample.

**Figure S1 Distribution of the Frailty Index in the studied population**


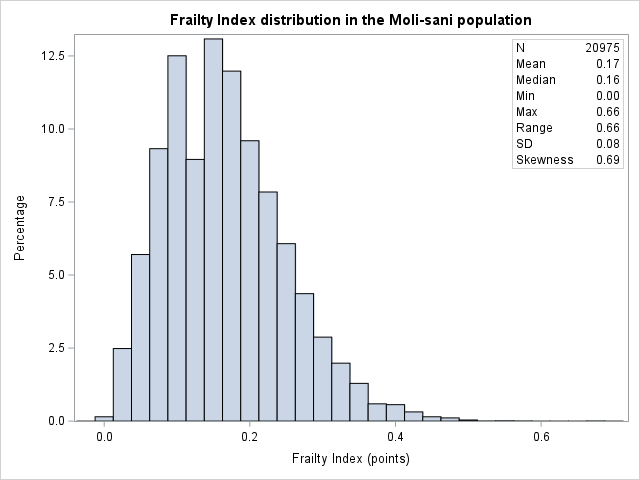


**Figure S2 Frailty Index as Continuous Exposure and Risk of Mortality: Spline Analysis**

**
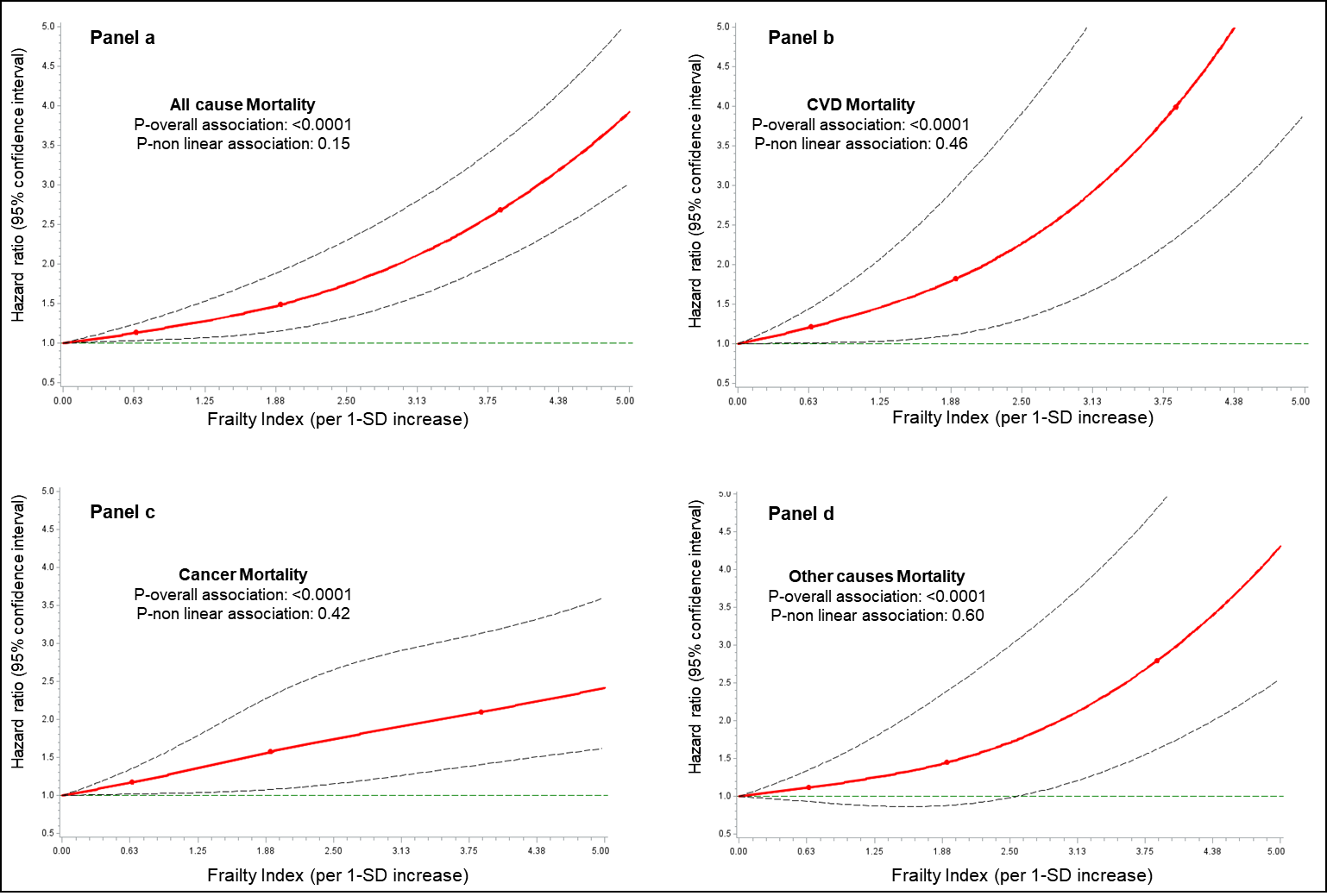
**

Panels a to d show mortality from all causes, cardiovascular disease, cancer, and other causes, respectively. Analyses adjusted for age, sex, smoking status, educational level, housing status, adherence to the Mediterranean diet, and leisure-time physical activity and competing risks were accounted for in domain-specific outcomes. Points represent knots at the 5th, 50th, and 95th percentiles. The reference value is set at FI=0.0. Dotted lines indicate 95% confidence intervals.

**Figure S3** **Frailty Index and Risk of Outcomes: Sensitivity Analyses Excluding Short Follow-up (≤1 and ≤3 Years)**


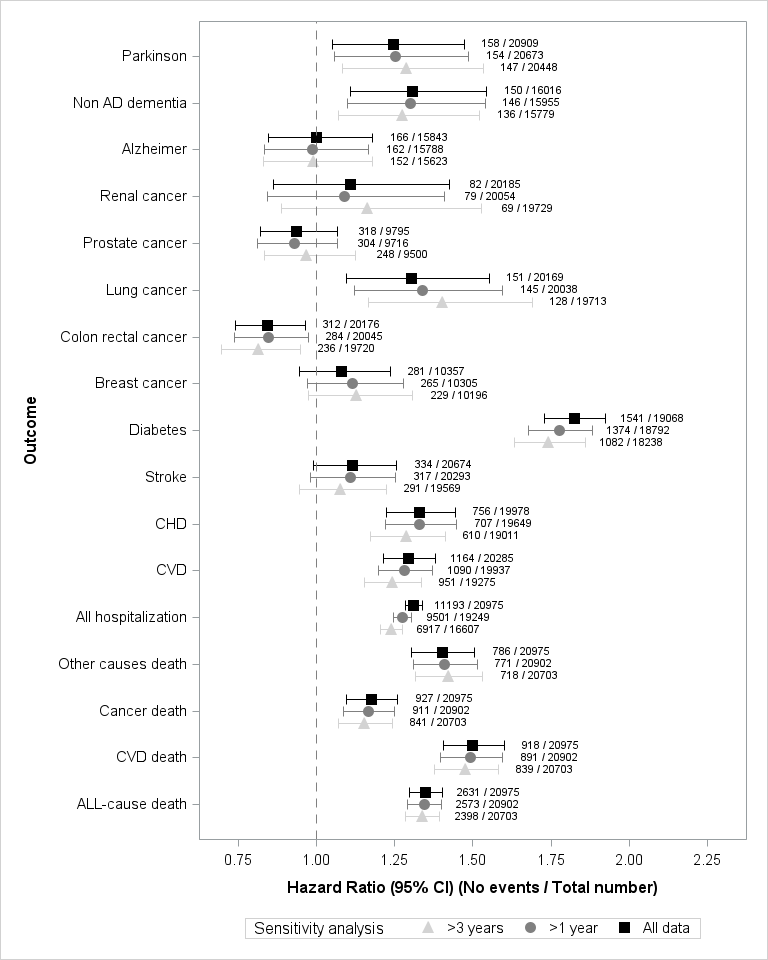


Analyses adjusted for age, sex, smoking status, educational level, housing status, adherence to the Mediterranean diet, and leisure-time physical activity and competing risks were accounted for in domain-specific outcomes. Horizontal bars represent 95% confidence intervals. For each outcome, hazard ratios are shown for the full population and after excluding individuals with ≤1 or ≤3 years of follow-up. Numbers at the lower right indicate events and population at risk within each subsample.

**Figure S4 Frailty Index and Risk of Outcomes: Subgroups Analysis in Men and Women**


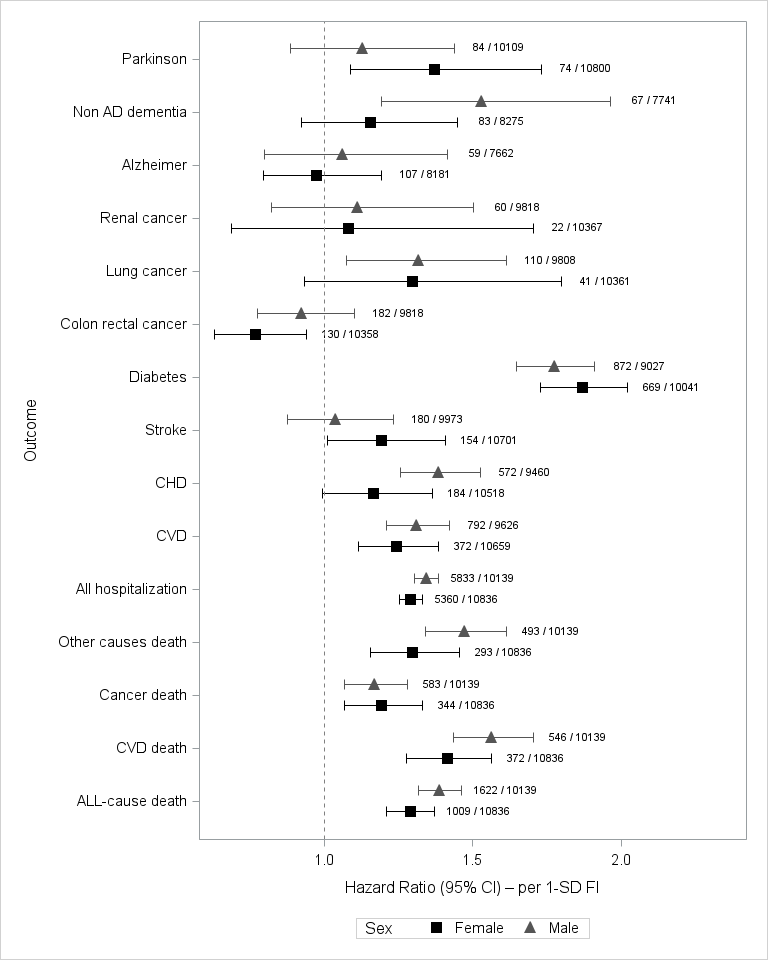


Analyses adjusted for age, smoking status, educational level, housing status, adherence to the Mediterranean diet, and leisure-time physical activity and competing risks were accounted for in domain-specific outcomes. Horizontal bars represent 95% confidence intervals. For each outcome, hazard ratios are shown for men and women. Numbers at the lower right indicate events and population at risk within each subsample. The p-value for interaction between frailty and sex was >0.05 for all outcomes except for all-cause hospitalization (p<0.001).

**Appendix 3**

**Moli-sani Study Investigators**
The enrolment phase of the Moli-sani Study was conducted at the Research Laboratories of the Catholic University in Campobasso (Italy), the follow up of the Moli-sani cohort is being conducted at the Research Unit of Epidemiology and Prevention of the IRCCS Neuromed, Pozzilli, Italy.
Steering Committee: Licia Iacoviello*# (chairperson), Giovanni de Gaetano* and Maria Benedetta Donati*.
Scientific Secretariat: Chiara Cerletti* (coordinator), Marialaura Bonaccio*, Americo Bonanni*, Simona Costanzo*°, Amalia De Curtis*, Augusto Di Castelnuovo*, Alessandro Gialluisi*#, Francesco Gianfagna°, Mariarosaria Persichillo*, Teresa Di Prospero* (secretary).
Safety and Ethical Committee: Jos Vermylen (Catholic University, Leuven, Belgium) (Chairperson), Renzo Pegoraro (Pontificia Accademia per la Vita, Vatican City), Antonio Spagnolo (Catholic University, Roma, Italy).
External Event Adjudicating Committee: Deodato Assanelli (Brescia, Italy), Livia Rago (Campobasso, Italy).
Baseline and Follow-up Data Management: Simona Costanzo*° (coordinator), Marco Olivieri (Campobasso, Italy), Sabatino Orlandi*, Teresa Panzera*.
Data Analysis: Augusto Di Castelnuovo* (coordinator), Marialaura Bonaccio*, Simona Costanzo*°, Simona Esposito*, Alessandro Gialluisi*#, Anwal Ghulam°, Francesco Gianfagna°, Antonietta Pepe*, Emilia Ruggiero*, Francesca Bracone*, Sukshma Sharma*.
Biobank, Molecular and Genetic Laboratory: Amalia De Curtis* (Coordinator), Concetta Civitillo*†, Alisia Cretella*†, Sara Magnacca*, Fabrizia Noro*.
Recruitment Staff: Mariarosaria Persichillo* (coordinator), Francesca Bracone*, Giuseppe Di Costanzo*, Fiorella De Rita (Cuore Sano ETS, Campobasso), Martina Morelli*†, Teresa Panzera*, Fiorella De Rito*.
Communication and Press Office: Americo Bonanni*.
Regional Institutions: Direzione Generale per la Salute - Regione Molise; Azienda Sanitaria Regionale del Molise (ASReM, Italy); Agenzia Regionale per la Protezione Ambientale del Molise (ARPA Molise, Italy); Molise Dati Spa (Campobasso, Italy); Offices of vital statistics of the Molise region.
Hospitals: Presidi Ospedalieri ASReM: Ospedale A. Cardarelli – Campobasso, Ospedale F. Veneziale – Isernia, Ospedale San Timoteo - Termoli (CB), Ospedale Ss. Rosario - Venafro (IS), Ospedale Vietri – Larino (CB), Ospedale San Francesco Caracciolo - Agnone (IS); Casa di Cura Villa Maria - Campobasso; Responsible Research Hospital - Campobasso; IRCCS Neuromed - Pozzilli (IS).
*Unit of Epidemiology and Prevention, IRCCS Neuromed, Pozzilli, Italy
#Department of Medicine and Surgery, LUM University “Giuseppe Degennaro”, Casamassima, Italy
°Department of Medicine and Surgery, University of Insubria, Varese, Italy
†Fondazione Veronesi – Piattaforma UMBERTO
Moli-sani Study Past Investigators are available at <https://www.moli-sani.org/?page_id=173>
